# Vibration characteristics of an active mounting system for motion control of a plate-like structure in future mobilities

**DOI:** 10.1038/s41598-023-43419-w

**Published:** 2023-09-28

**Authors:** Dongwoo Hong, Yang Qiu, Byeongil Kim

**Affiliations:** 1 Daegu Mechatronics & Materials Institute, 32, Seongseogongdan-ro 11-gil, Dalseo-gu, Daegu, 42714 Republic of Korea; 2 School of Mechanical Engineering, Yeungnam University, Gyeongsan, 38541 Republic of Korea

**Keywords:** Engineering, Mechanical engineering

## Abstract

The vibration and noise caused by electric motors in hybrid and electric vehicles (EVs) generate complex signals with a mid-frequency band, which causes uncomfortable vibration and noise. In order to isolate the vibration and noise, active engine mounting systems based on smart structures have attracted attention. Thus, in this study, the vibration attenuation performance was validated through simulation and feasibility experiments by applying an active mounting system using a piezoelectric stack actuator. A plate structure with three paths, consisting of two passive paths and one active path, was modeled using the lumped parameter method. The source part was excited by a sinusoidal and modulated signal with a mid-frequency band to validate the vibration attenuation performance. Furthermore, (1) mathematical modeling with a source-path-receiver structure was proposed based on lumped parameter modeling, (2) normalized least mean square (NLMS) and multi-NLMS algorithms were applied to implement motion control, and (3) a principal experimental setup was designed to validate the simulation results. Through this process, the vibration attenuation performance of the proposed active mount structure was validated.

## Introduction

Recently, hybrid and electric vehicles (EVs) have become increasingly popular. Because vibrations and noise caused by electric motors have a complex spectrum, they cause discomfort to the driver while driving and cannot be neglected. In addition, to enhance the performance of EVs, car manufacturers reduce the weight of the vehicle body and increase the engine output, which increases the complex vibrations. Owing to these issues, traditional noise, vibration, and harshness (NVH) development skills have limitations, and an active engine-mounting system based on a smart structure has attracted attention. By implementing the static and dynamic stiffnesses of the engine and continuously controlling the dynamic characteristics of the mount, an active mounting system can dramatically improve the NVH performance of an EV. Active noise and vibration control using smart materials has been actively explored and devoted to vibration attenuation by applying an active mounting system.

To control the vibration, adaptive control algorithms, such as the filtered-x least mean square (FX-LMS) algorithm^1^, sliding mode control^2^, and notch filtered algorithm^3^, are widely applied. Fakhari et al. conducted vibration isolation using an electromagnetic engine mount by applying robust model reference adaptive control (MRAC)^4^. Kim et al. focused on an adaptive control algorithm to improve LMS algorithm performance^5–7^. They applied sliding mode control, and experiments were conducted using an active strut structure. In addition, model predictive sliding mode control (MPSMC) has been proposed to compensate for the defects in adaptive filtering algorithms. The proposed algorithm was verified for efficient vibration reduction performance through experiments using a strut structure and cantilevered beam. The applied and developed adaptive algorithm in the literature achieved a good performance when applied to smart structure-based active mounting systems.

An active mounting system using various smart structures, such as solenoids^8^, integrated magneto-rheological fluids and piezoelectric stack actuators^9^, and electrorheological fluid^10, 11^, has been proposed to attenuate the vibration of the engine motor. Hausberg et al. analyzed the nonlinearity, temperature, and preload change through models of and experiments on active engine mounts. Furthermore, to characterize the active mounting system, Harun et al. analyzed the characterization of a magnetorheological fluid (MR) damper applied in active engine mounting. The experiment demonstrated that the MR damper is suitable for active engine mounting^12^. Wu et al. developed a vibration isolator by applying the characteristics of a magnetic spring with a negative stiffness^13^. This indicated that the application of the proposed method reduced the natural frequency. Truong et al. proposed a mathematical model for a novel hydraulic engine mount^14^. The simulation results confirmed that the resonance peak and natural frequency could be changed through optimal tuning. Kamada et al. combined a column structure and piezoelectric actuator and showed that structural vibration can be efficiently reduced^15^. Loukil et al. proposed a method for supplying a piezo actuator through energy harvesting and found that vibration can be effectively reduced using energy harvesting^16^. Sui et al. manufactured a vehicle engine mount using PZT and validated that the proposed method can reduce vibration of the mount^17^. Bartel et al. proposed a novel type of engine mount that was designed to withstand dynamic forces^18^. The results revealed that the proposed engine mount could be used for vibration isolation. Liette et al. conducted studies to quantify the active path when a structure was excited by a single frequency^19^. The results revealed that source motion could be isolated by applying the calculated quantification parameters. Although the proposed method exhibited great performance, it had only been carried out in simulations and experiments with a simple model and signal. However, in the real world, the actual vehicle engine consists of three to four mounts and undergoes rotational movements such as rolling, pitching, and yawing. Furthermore, vibrations generated by electric motors have a complex spectrum in the mid-frequency range, which is not typically observed in conventional ICEs. Thus, the efficiency of active mounting systems should be validated using more complex models and signals. Liette at al. assumed that all the motions occurring in the engine are simplified, and the proposed quantification method has a limitation when complex signals are considered. To cover a more detailed vibration effect and overcome the limitations of the quantification method, the proposed model should be expanded, and a better algorithm should be applied. Furthermore, in the case of MR or ER fluid, which is currently being studied, it has excellent performance in controlling vibration in the low frequency band generated from ICEs. However, it is not suitable for controlling vibration in the mid-frequency range generated by electric motors. Therefore, in this paper, we focused on the electric vehicle, which is currently actively being researched and developed, and proposed an active mount system by applying a piezoelectric actuator capable of precise position control for mid-frequency control generated by an electric motor.

**Figure 1:**
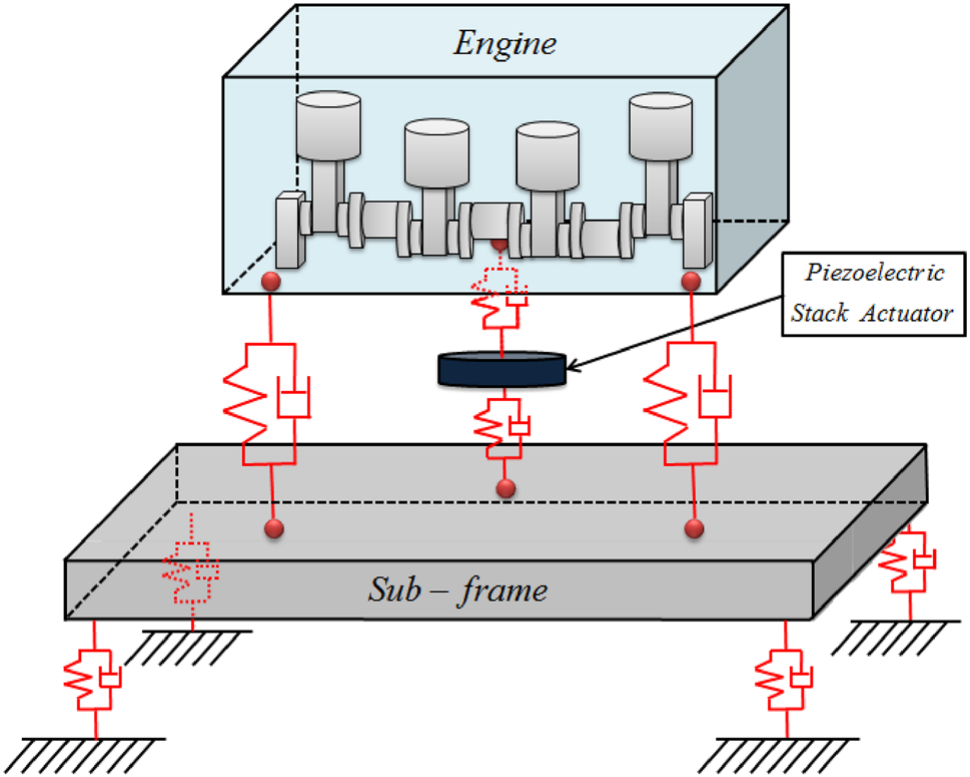
Powertrain with three paths.

To validate the vibration attenuation performance through a novel active mounting system, a powertrain motivated structural system including an active component is proposed for analysis purposes, as shown in Fig. 1. It is based on the source-path-receiver structure: the source part represents the engine, and the receiver part represents the sub-frame. Furthermore, it includes two passive paths and one active path. The passive paths are only composed of rubber grommets, and the active path consists of a piezoelectric stack actuator and rubber grommet. In addition, to validate the performance of vibration attenuation, a numerical simulation and feasibility experiment were conducted, in which the source part was excited by sinusoids and amplitude modulated (AM) signals with mid-frequency bands. To verify the vibration reduction performance, the actuator input was defined by applying a mathematical quantification method and NLMS algorithm^20^. The reason for selecting the NLMS algorithm is as follows. Since NLMS has a simple structure, it has the advantage of being easy to implement. In particular, it has excellent performance in tracking signals compared to simple structures, so it is widely applied in many industrial fields to reduce plant vibration and noise. Furthermore, in order to track signals with multiple frequencies, multiple NLMS algorithms are actively used. The main contributions of this study are as follows: (1) A lumped parameter modeling method that can consider all x, y, and z directions was proposed, and based on this method, mathematical modeling of a structure with an active mounting system was performed and motion equations were derived.; (2) Based on the modeling presented in 1), the input of the actuator was quantified and the vibration reduction performance was verified; (3) Furthermore, the limitations of the quantification method were suggested, and the vibration reduction performance was compared when the NLMS algorithm and Multi-NLMS were applied; and (4) to validate the simulation results, a feasibility experiment was conducted using a principal experimental setup, and the vibration-reduction performance is discussed.

The system was assumed to be deterministic, frequency-independent, and discrete. In addition, it was considered a linear system without nonlinear kinematic effects, such as the hysteresis effect in the actuator, indicating that the superposition principle was applicable. The source and receiver parts were assumed to be rigid, without flexural participation. In addition, when the disturbance force was considered, the known harmonic perturbation force excited the source. To control the source motion, an adaptive algorithm was applied to an active mounting system. In the modeling of the active structural system, the mass of the rubber grommet was ignored, while the mass of the active component was not. In addition, based on the Kelvin–Voigt model, the damping coefficient was considered using complex stiffness values. Finally, when structural movement was considered, the structure had only vertical displacement, and displacements in other directions (*y* and *z* directions) were ignored.

The remainder of this paper is organized as follows. In “Mounting system modeling” section, the mathematical modelling of a structure with an active mounting system is described. In “Quantification of actuator input” section, quantification of the actuator force and phase is summarized. In “Numerical performance validation with a single sinusoid” section, the performance of vibration attenuation between the quantification method and the NLMS algorithm is described. In “Numerical performance validation with a multi-spectral signal” section, when the AM signal is used as the disturbance force, the vibration attenuation performance is discussed by applying the multi-NLMS algorithm. In “Experimental validation” section, a description of the feasibility experiment conducted using the principle experimental setup to validate the simulation trend is provided, and the results are discussed. Finally, in “Conclusion” section, conclusions and future work are summarized.

## Mounting system modeling

**Figure 2:**
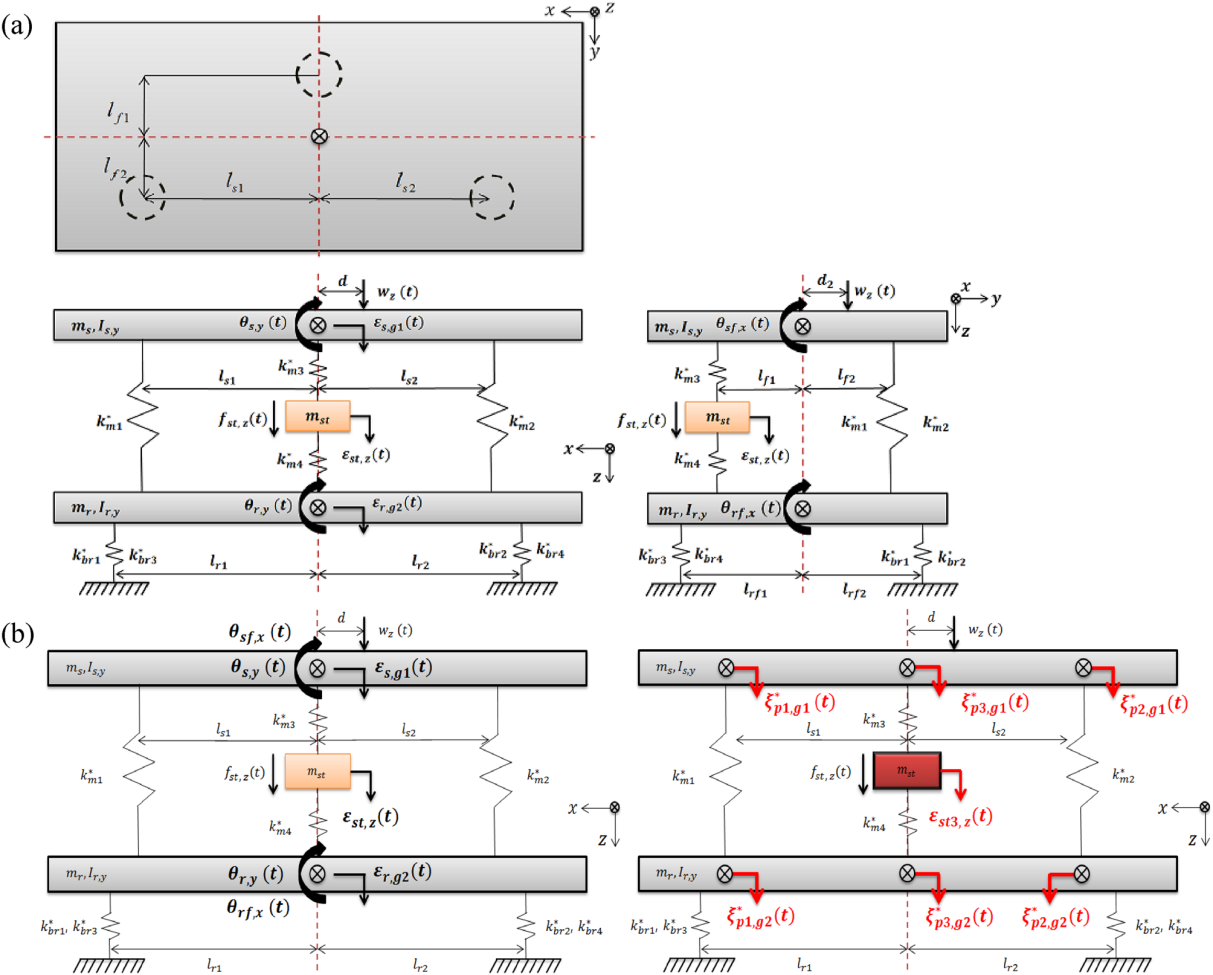
(**a**) Modeling of a plate with three mounting positions, (**b**) coordinate transformation (left: centers of masses, right: path locations).

To derive the equation of motion for an active structural system, lumped parameter modelling was performed, as shown in Fig. 2a. Path 1 and path 2 consist of only a rubber grommet, and path 3 consists of a piezoelectric stack actuator and rubber grommet, which means that path 3 is an active path.

In Fig. 2a, ${m}_{s}$ and ${m}_{r}$ represent the masses corresponding to the source and receiver, respectively. ${m}_{st}$ represents the actuator mass. Through measurements, a realistic value for the mass was obtained and used. ${I}_{s,y}$ and ${I}_{r,y}$ represent the moments of inertia in the y-direction, corresponding to the source and receiver, respectively. In addition, ${I}_{sf,x}$ and ${I}_{rf,x}$ represent the moments of inertia in the x-direction corresponding to the source and receiver, respectively. The moment of inertia was calculated and used. ${l}_{si}$ and ${l}_{ri}$ represent the lengths corresponding to the center of mass and each mount in the front view, respectively. ${l}_{fi}$ and ${l}_{rfi}$ represent the lengths corresponding to the side view. $d$ and ${d}_{2}$ represent the distance from the center of the mass to the shaker. ${k}_{mi}^{*}$ and ${k}_{bri}^{*}$ represent the complex stiffness in each path between the source and receiver and between the receiver and ground, respectively. All parameters were measured and calculated from the laboratory setup constructed for this study (refer to “Experimental validation” section), and these values are described in Table 1.

**Table 1:** Parameter values and units for the given model.

Parameter	Value	Unit	Parameter	Value	Unit
${m}_{s}={m}_{r}$	$1.081$	$\mathrm{kg}$	${k}_{br1}^{*}={k}_{br2}^{*}$	$0.42(1+i0.300)$	kN mm^−1^
${m}_{st}$	$0.075$	$\mathrm{kg}$	${k}_{br3}^{*}={k}_{br4}^{*}$	$0.42(1+i0.300)$	kN mm^−1^
${I}_{s,y}={I}_{r,y}$	$19.44$	gm^2^	${l}_{s1}={l}_{s2}$	$100$	mm
${I}_{sf,x}={I}_{rf,x}$	$10.29$	gm^2^	${l}_{r1}={l}_{r2}$	$140$	mm
${k}_{m1}^{*}$	$0.610(1+i0.034)$	kN mm^−1^	${l}_{f1}={l}_{f2}$	$80$	mm
${k}_{m2}^{*}$	$0.610(1+i0.034)$	kN mm^−1^	${l}_{rf1}={l}_{rf2}={l}_{rf3}={l}_{rf4}$	$100$	mm
${k}_{m3}^{*}$	$5.640(1+i0.036)$	kN mm^−1^	$d$	$50$	mm
${k}_{m4}^{*}$	$0.530(1+i0.300)$	kN mm^−1^	${d}_{2}$	$10$	mm

${\varepsilon }_{s,g1}$, ${\varepsilon }_{r,g2}$, and ${\varepsilon }_{st}$ represent the displacements in the *z*-direction corresponding to the source, receiver, and actuator, respectively. ${\theta }_{s,y}$ and ${\theta }_{r,y}$ represent the rotational displacements in the *y*-direction, corresponding to the source and receiver, respectively. ${\theta }_{sf,x}$ and ${\theta }_{rf,x}$ represent the rotational displacements in the *x*-direction corresponding to each plate. ${f}_{st}$ and ${w}_{z}$ represent the forces corresponding to the actuator and the shaker, respectively. To conduct this study, the following assumptions were made: (1) Translational motion was considered only in the *z*-direction; (2) the rotational motions in each view, as well as the front and side, were considered to be only in the *y*- and *x*-directions, respectively; (3) the hysteresis phenomena of the actuator were ignored. Based on Fig. 2a, the overall equation of motion was derived, as shown in Eqs. (1)–(7).

1 $$\begin{aligned} & {m}_{s}{\ddot{\varepsilon }}_{s,g1}+\left({k}_{m1}^{*}+{k}_{m3}^{*}+{k}_{m2}^{*}\right){\varepsilon }_{s,g1}+\left(\begin{array}{c}-{k}_{m1}^{*}\\ -{k}_{m2}^{*}\end{array}\right){\varepsilon }_{r,g1}-{k}_{m3}^{*}{\varepsilon }_{st}+\left(\begin{array}{c}{k}_{m2}^{*}{l}_{s2}\\ -{k}_{m1}^{*}{\varepsilon }_{s1}\end{array}\right){\theta }_{s,y} \\ & \quad \quad +\left(\begin{array}{c}{k}_{m1}^{*}{l}_{s1}\\ -{k}_{m2}^{*}{\varepsilon }_{s2}\end{array}\right){\theta }_{r,y}+\left({k}_{m1}^{*}{l}_{f2}+{k}_{m2}^{*}{l}_{f2}-{k}_{m3}^{*}{l}_{f1}\right){\theta }_{sf,x}+\left(\begin{array}{c}-{k}_{m1}^{*}{l}_{f2}\\ -{k}_{m2}^{*}{l}_{f2}\end{array}\right){\theta }_{rf,x}={w}_{z}(t)\end{aligned}$$

2 $$\begin{aligned} & {m}_{r}{\ddot{\varepsilon }}_{r,g1}+\left(\begin{array}{c}-{k}_{m1}^{*}\\ -{k}_{m2}^{*}\end{array}\right){\varepsilon }_{s,g1}+\left({k}_{m1}^{*}+{k}_{m2}^{*}+{k}_{m4}^{*}+{k}_{br1}^{*}+{k}_{br2}^{*}+{k}_{br3}^{*}+{k}_{br4}^{*}\right){\varepsilon }_{r,g1}-{k}_{m4}^{*}{\varepsilon }_{st} \\ & \quad \quad +\left(\begin{array}{c}{k}_{m1}^{*}{l}_{s1}\\ -{k}_{m2}^{*}{l}_{s2}\end{array}\right){\theta }_{s,y}+\left({k}_{m1}^{*}{l}_{s2}-{k}_{m2}^{*}{l}_{s1}-{k}_{br1}^{*}{l}_{r1}+{k}_{br2}^{*}{l}_{r2}-{k}_{br3}^{*}{l}_{r1}+{k}_{br4}^{*}{l}_{r2}\right){\theta }_{r,y} \\ & \quad \quad +\left(\begin{array}{c}-{k}_{m1}^{*}{l}_{f2}\\ -{k}_{m2}^{*}{l}_{f2}\end{array}\right){\theta }_{sf,x}+({k}_{m1}^{*}{l}_{f2}+{k}_{m2}^{*}{l}_{f2}-{k}_{m4}^{*}{l}_{f1}+{k}_{br1}^{*}{l}_{rf2}+{k}_{br2}^{*}{l}_{rf2}-{k}_{br3}^{*}{l}_{rf1}-{k}_{br4}^{*}{l}_{rf1}){\theta }_{rf,x}=0\end{aligned}$$

3 $${m}_{st}{\ddot{\varepsilon }}_{st,g1}+\left(\begin{array}{c}{k}_{m3}^{*}\\ {k}_{m4}^{*}\end{array}\right){\varepsilon }_{st}-{k}_{m3}^{*}{\varepsilon }_{s,g1}-{k}_{m4}^{*}{\varepsilon }_{r,g1}+{k}_{m3}^{*}{l}_{f1}{\theta }_{sf,x}+{k}_{m4}^{*}{l}_{f1}{\theta }_{rf,x}={f}_{st}(t)$$

4 $${I}_{s}{\ddot{\theta }}_{s,y}+\left(\begin{array}{c}{k}_{m2}^{*}{l}_{s2}\\ -{k}_{m1}^{*}{l}_{s1}\end{array}\right){\varepsilon }_{s,g1}+\left(\begin{array}{c}{k}_{m1}^{*}{l}_{s1}\\ -{k}_{m2}^{*}{l}_{s2}\end{array}\right){\varepsilon }_{r,g1}+\left(\begin{array}{c}{k}_{m1}^{*}{l}_{s1}^{2}\\ {k}_{m2}^{*}{l}_{s2}^{2}\end{array}\right){\theta }_{s,y}+\left(\begin{array}{c}-{k}_{m1}^{*}{l}_{s1}^{2}\\ -{k}_{m2}^{*}{l}_{s2}^{2}\end{array}\right){\theta }_{r,y}={w}_{z}(t)d$$

5 $$\begin{aligned} & {I}_{r}{\ddot{\theta }}_{r,y}+\left(\begin{array}{c}{k}_{m1}^{*}{l}_{s1}\\ -{k}_{m2}^{*}{l}_{s2}\end{array}\right){\varepsilon }_{s,g1}+\left({k}_{m1}^{*}{l}_{s2}-{k}_{m2}^{*}{l}_{s1}-{k}_{br1}^{*}{l}_{r1}+{k}_{br2}^{*}{l}_{r2}-{k}_{br3}^{*}{l}_{r1}+{k}_{br4}^{*}{l}_{r2}\right){\varepsilon }_{r,g1} \\ & \quad \quad +\left(\begin{array}{c}-{k}_{m1}^{*}{l}_{s1}^{2}\\ -{k}_{m2}^{*}{l}_{s2}^{2}\end{array}\right){\theta }_{s,y}+({k}_{m1}^{*}{l}_{s1}^{2}+{k}_{m2}^{*}{l}_{s2}^{2}+{k}_{br1}^{*}{l}_{r1}^{2}+{k}_{br2}^{*}{l}_{r1}^{2}+{k}_{br3}^{*}{l}_{r2}^{2}+{k}_{br4}^{*}{l}_{r2}^{2}){\theta }_{r,y}=0\end{aligned}$$

6 $$\begin{aligned} & {I}_{sf}{\ddot{\theta }}_{sf,y}+\left({k}_{m1}^{*}{l}_{f2}+{k}_{m2}^{*}{l}_{f2}-{k}_{m3}^{*}{l}_{f1}\right){\varepsilon }_{s,g1}+\left(\begin{array}{c}-{k}_{m1}^{*}{l}_{f2}\\ -{k}_{m2}^{*}{l}_{f2}\end{array}\right){\varepsilon }_{r,g1}+{k}_{m3}^{*}{l}_{f1}{\varepsilon }_{st} \\ & \quad \quad +({k}_{m1}^{*}{l}_{f2}^{2}+{k}_{m2}^{*}{l}_{s2}^{2}+{k}_{m3}^{*}{l}_{s1}^{2}){\theta }_{sf,x}+\left(\begin{array}{c}-{k}_{m1}^{*}{l}_{f2}^{2}\\ -{k}_{m2}^{*}{l}_{s2}^{2}\end{array}\right){\theta }_{rf,x}={w}_{z}(t){d}_{2} \end{aligned}$$

7 $$\begin{aligned} & {I}_{rf}{\ddot{\theta }}_{rf,y}+\left(\begin{array}{c}-{k}_{m1}^{*}{l}_{f2}\\ -{k}_{m2}^{*}{l}_{f2}\end{array}\right){\varepsilon }_{s,g1}+\left({k}_{m1}^{*}{l}_{f2}+{k}_{m2}^{*}{l}_{f2}-{k}_{m4}^{*}{l}_{f1}+{k}_{br1}^{*}{l}_{f2}+{k}_{br2}^{*}{l}_{f2}-{k}_{br3}^{*}{l}_{f1}-{k}_{br4}^{*}{l}_{f1}\right){\varepsilon }_{r,g1}+{k}_{m4}^{*}{l}_{f1}{\varepsilon }_{st} \\ & \quad \quad +\left(\begin{array}{c}-{k}_{m1}^{*}{l}_{f2}^{2}\\ -{k}_{m2}^{*}{l}_{f2}^{2}\end{array}\right){\theta }_{sf,x}+({k}_{m1}^{*}{l}_{f2}^{2}+{k}_{m2}^{*}{l}_{f2}^{2}+{k}_{m4}^{*}{l}_{f1}^{2}+{k}_{br1}^{*}{l}_{f2}^{2}+{k}_{br2}^{*}{l}_{f2}^{2}+{k}_{br3}^{*}{l}_{f1}^{2}+{k}_{br4}^{*}{l}_{f1}^{2}){\theta }_{rf,y}=0\end{aligned}$$

To simplify the equation of motion, it can be summarized in matrix form, as shown by Eqs. (8)–(12), where ${\varvec{M}}$ represents the mass matrix, ${{\varvec{K}}}^{*}$ represents the complex stiffness matrix, ${\varvec{q}}(t)$ represents the displacement matrix, and ${\varvec{w}}\left(t\right)$ and ${\varvec{F}}(t)$ represent the force matrices corresponding to the disturbance and actuator, respectively.

8 $${\varvec{M}}={\varvec{d}}{\varvec{i}}{\varvec{a}}{\varvec{g}}(\{{{\varvec{m}}}_{{\varvec{s}}} {{\varvec{m}}}_{{\varvec{r}}} {{\varvec{m}}}_{{\varvec{s}}{\varvec{t}}} {{\varvec{I}}}_{{\varvec{s}},{\varvec{y}}} {{\varvec{I}}}_{{\varvec{r}},{\varvec{y}}} {{\varvec{I}}}_{{\varvec{s}}{\varvec{f}},{\varvec{y}}} {{\varvec{I}}}_{{\varvec{r}}{\varvec{f}},{\varvec{x}}}\})$$

9 $${\varvec{q}}\left(t\right)=\left\{\begin{array}{ccc}{\varepsilon }_{s,g1}(t)& {\varepsilon }_{r,g2}(t)& \begin{array}{ccc}{\varepsilon }_{st}(t)& {\theta }_{s,y}(t)& \begin{array}{ccc}{\theta }_{r,y}(t)& {\theta }_{sf,x}(t)& {\theta }_{rf,x}(t)\end{array}\end{array}\end{array}\right\}$$

10 $${\varvec{w}}\left(t\right)=\left\{\begin{array}{ccc}{w}_{z}(t)& 0& \begin{array}{ccc}0& {w}_{z}(t)d& \begin{array}{ccc}0& {w}_{z}\left(t\right){d}_{2}& 0\end{array}\end{array}\end{array}\right\}$$

11 $${\varvec{F}}\left(t\right)=\left\{\begin{array}{ccc}0& 0& \begin{array}{ccc}{f}_{st}(t)& 0& \begin{array}{ccc}0& 0& 0\end{array}\end{array}\end{array}\right\}$$

12 $${K}^{*}=\left[\begin{array}{cccc}{k}_{m1}^{*}+{k}_{m2}^{*}+{k}_{m3}^{*}& -{k}_{m1}^{*}-{k}_{m2}^{*}& -{k}_{m3}^{*}& {k}_{m2}^{*}{l}_{s2}-{k}_{m1}^{*}{l}_{s1}\\ -{k}_{m1}^{*}-{k}_{m2}^{*}& {k}_{m1}^{*}+{k}_{m2}^{*}+{k}_{m4}^{*}+{k}_{br1}^{*}+{k}_{br2}^{*}+{k}_{br3}^{*}+{k}_{br4}^{*}& -{k}_{m4}^{*}& {k}_{m1}^{*}{l}_{s1}-{k}_{m2}^{*}{l}_{s2}\\ -{k}_{m3}^{*}& -{k}_{m4}^{*}& {k}_{m3}^{*}+{k}_{m4}^{*}& 0\\ {k}_{m2}^{*}{l}_{s2}-{k}_{m1}^{*}{l}_{s1}& {k}_{m1}^{*}{l}_{s1}-{k}_{m2}^{*}{l}_{s2}& 0& {k}_{m1}^{*}{l}_{s1}^{2}+{k}_{m2}^{*}{l}_{s2}^{2}\\ {k}_{m1}^{*}{l}_{s1}-{k}_{m2}^{*}{l}_{s2}& {k}_{m1}^{*}{l}_{s2}-{k}_{m2}^{*}{l}_{s1}-{k}_{br1}^{*}{l}_{r1}+{k}_{br2}^{*}{l}_{r2}-{k}_{br3}^{*}{l}_{r1}+{k}_{br4}^{*}{l}_{r2}& 0& -{k}_{m1}^{*}{l}_{s1}^{2}-{k}_{m2}^{*}{l}_{s2}^{2}\\ {k}_{m1}^{*}{l}_{f2}+{k}_{m2}^{*}{l}_{f2}-{k}_{m3}^{*}{l}_{f1}& -{k}_{m1}^{*}{l}_{f2}-{k}_{m2}^{*}{l}_{f2}& {k}_{m3}^{*}{l}_{f1}& 0\\ -{k}_{m1}^{*}{l}_{f2}-{k}_{m2}^{*}{l}_{f2}& {k}_{m1}^{*}{l}_{f2}+{k}_{m2}^{*}{l}_{f2}-{k}_{m4}^{*}{l}_{f1}+{k}_{br1}^{*}{l}_{fr2}+{k}_{br2}^{*}{l}_{fr2}-{k}_{br3}^{*}{l}_{fr1}-{k}_{br4}^{*}{l}_{fr1}& {k}_{m4}^{*}{l}_{f1}& 0\end{array}\right.$$

$$\left.\begin{array}{ccc}{k}_{m1}^{*}{l}_{s1}-{k}_{m2}^{*}{l}_{s2}& {k}_{m1}^{*}{l}_{f2}+{k}_{m2}^{*}{l}_{f2}-{k}_{m3}^{*}{l}_{f1}& -{k}_{m1}^{*}{l}_{f2}-{k}_{m2}^{*}{l}_{f2}\\ {k}_{m1}^{*}{l}_{s2}-{k}_{m2}^{*}{l}_{s1}-{k}_{br1}^{*}{l}_{r1}+{k}_{br2}^{*}{l}_{r2}-{k}_{br3}^{*}{l}_{r1}+{k}_{br4}^{*}{l}_{r2}& -{k}_{m1}^{*}{l}_{f2}-{k}_{m2}^{*}{l}_{f2}& {k}_{m1}^{*}{l}_{f2}+{k}_{m2}^{*}{l}_{f2}-{k}_{m4}^{*}{l}_{f1}+{k}_{br1}^{*}{l}_{fr2}+{k}_{br2}^{*}{l}_{fr2}-{k}_{br3}^{*}{l}_{fr1}-{k}_{br4}^{*}{l}_{fr1}\\ 0& {k}_{m3}^{*}{l}_{f1}& {k}_{m4}^{*}{l}_{f1}\\ -{k}_{m1}^{*}{l}_{s1}-{k}_{m2}^{*}{l}_{s2}& 0& 0\\ {k}_{m1}^{*}{l}_{s2}^{2}+{k}_{m2}^{*}{l}_{s2}^{2}+{k}_{br1}^{*}{l}_{r1}^{2}+{k}_{br2}^{*}{l}_{r1}^{2}+{k}_{br3}^{*}{l}_{r2}^{2}+{k}_{br4}^{*}{l}_{r2}^{2}& 0& 0\\ 0& {k}_{m1}^{*}{l}_{f2}^{2}+{k}_{m2}^{*}{l}_{f2}^{2}+{k}_{m3}^{*}{l}_{f1}^{2}& -{k}_{m1}^{*}{l}_{f2}^{2}-{k}_{m2}^{*}{l}_{f2}^{2}\\ 0& -{k}_{m1}^{*}{l}_{f2}^{2}-{k}_{m2}^{*}{l}_{f2}^{2}& {k}_{m1}^{*}{l}_{f2}^{2}+{k}_{m2}^{*}{l}_{f2}^{2}+{k}_{m4}^{*}{l}_{f1}^{2}+{k}_{br1}^{*}{l}_{fr2}^{2}+{k}_{br2}^{*}{l}_{fr2}^{2}+{k}_{br3}^{*}{l}_{fr1}^{2}+{k}_{br4}^{*}{l}_{fr1}^{2}\end{array}\right]$$

In this study, we focused on the vibration attenuation performance of a path that includes an active mounting system. Thus, the coordinates for each path location should be used in the equation of motion. The transfer matrix $\Pi$ was, therefore, defined to achieve this, as shown in Eq. (13).

13 $$\prod =\left[\begin{array}{ccccccc}\frac{{l}_{s2}*{l}_{f1}}{\left({l}_{s1}+{l}_{s2}\right)\left({l}_{f1}+{l}_{f2}\right)}& \frac{{l}_{s1}*{l}_{f1}}{\left({l}_{s1}+{l}_{s2}\right)\left({l}_{f1}+{l}_{f2}\right)}& \frac{{l}_{f2}}{{l}_{f1}+{l}_{f2}}& 0& 0& 0& 0\\ 0& 0& 0& 0& \frac{{l}_{s2}*{l}_{f1}}{\left({l}_{s1}+{l}_{s2}\right)\left({l}_{f1}+{l}_{f2}\right)}& \frac{{l}_{s1}*{l}_{f1}}{\left({l}_{s1}+{l}_{s2}\right)\left({l}_{f1}+{l}_{f2}\right)}& \frac{{l}_{f2}}{{l}_{f1}+{l}_{f2}}\\ 0& 0& 0& 1& 0& 0& 0\\ \frac{-1}{\left({l}_{s1}+{l}_{s2}\right)\left({l}_{f1}+{l}_{f2}\right)}& \frac{1}{\left({l}_{s1}+{l}_{s2}\right)\left({l}_{f1}+{l}_{f2}\right)}& 0& 0& 0& 0& 0\\ 0& 0& 0& 0& \frac{-1}{\left({l}_{s1}+{l}_{s2}\right)\left({l}_{f1}+{l}_{f2}\right)}& \frac{1}{\left({l}_{s1}+{l}_{s2}\right)\left({l}_{f1}+{l}_{f2}\right)}& 0\\ \frac{1}{\left({l}_{s1}+{l}_{s2}\right)\left({l}_{f1}+{l}_{f2}\right)}& \frac{1}{\left({l}_{s1}+{l}_{s2}\right)\left({l}_{f1}+{l}_{f2}\right)}& \frac{-1}{{l}_{s1}+{l}_{s2}}& 0& 0& 0& 0\\ 0& 0& 0& 0& \frac{1}{\left({l}_{s1}+{l}_{s2}\right)\left({l}_{f1}+{l}_{f2}\right)}& \frac{1}{\left({l}_{s1}+{l}_{s2}\right)\left({l}_{f1}+{l}_{f2}\right)}& \frac{-1}{{l}_{s1}+{l}_{s2}}\end{array}\right]$$

The definition of the transformed displacement is ${\varvec{q}}=\prod {{\varvec{q}}}^{\mathrm{^{\prime}}}$, where ${{\varvec{q}}}^{\mathrm{^{\prime}}}$ is defined in Eq. (14). Using Eq. (13), the equation of motion can be rewritten as Eq. (15), and Fig. 2b shows the transformed coordinates.

14 $${\varvec{q}}\mathrm{^{\prime}}\left(t\right)=\left\{\begin{array}{ccc}{\xi }_{p1,g1}(t)& {\xi }_{p2,g1}(t)& \begin{array}{ccc}{\xi }_{p3,g1}(t)& {\varepsilon }_{st}(t)& \begin{array}{ccc}{\xi }_{p1,g2}(t)& {\xi }_{p2,g2}(t)& {\xi }_{p3,g2}(t)\end{array}\end{array}\end{array}\right\}$$

15 $${\varvec{M}}\mathrm{^{\prime}}\ddot{{\varvec{q}}}\mathrm{^{\prime}}+{{\varvec{K}}}^{*}\mathrm{^{\prime}}{\varvec{q}}\mathrm{^{\prime}}={\varvec{F}}\left(t\right)+{\varvec{W}}(t)$$

## Quantification of actuator input

In this section, the actuator force and phase are quantified to isolate the vibrations of the target path. The results of the quantification method are compared with those obtained with the application of the NLMS algorithm, which is described in the next section. The disturbance and actuator force are defined by Eqs. (16) and (17), respectively:

16 $${w}_{z}\left(t\right)={W}_{z}{e}^{i\omega t};$$

17 $${f}_{st}\left(t\right)={F}_{st}{e}^{i\left(\omega t+{\phi }_{st}\right)}.$$

where ${W}_{z}$ and ${F}_{st}$ represent the amplitudes corresponding to the disturbance and the actuator, respectively. ${\phi }_{st}$ represents the phase that corresponds to the actuator. The motion according to the force is defined for each path as follows:

18 $${\xi }_{i}^{*}\left(t\right)={\xi }_{pi,g1}^{*}\left(t\right).$$

${\xi }_{pi,g1}^{*}(t)$ represents the motion corresponding to the *i*th path of inertia ${g}_{1}$ of the source part. The motion in each path is affected by the disturbance and actuator force. Thus, it can be expressed by Eq. (19).

19 $${\xi }_{i}^{*}(t)=({\Xi }_{si,1}^{*}+{\Xi }_{si,st}^{*}{e}^{i{\phi }_{st}}){e}^{i\omega t}$$

${\Xi }_{si,1}^{*}$ and ${\Xi }_{si,st}^{*}$ represent the amplitude corresponding to the effects of the disturbance and actuator force, respectively. To calculate the amplitude and phase, the compliance matrix is calculated to define the displacement caused by the unit load and is given by

20 $${{\varvec{H}}}^{*\mathrm{^{\prime}}}=\left[\begin{array}{ccccccc}{H}_{11}^{*\mathrm{^{\prime}}}& {H}_{12}^{*\mathrm{^{\prime}}}& {H}_{13}^{*\mathrm{^{\prime}}}& {H}_{14}^{*\mathrm{^{\prime}}}& {H}_{15}^{*\mathrm{^{\prime}}}& {H}_{16}^{*\mathrm{^{\prime}}}& {H}_{17}^{*\mathrm{^{\prime}}}\\ {H}_{21}^{*\mathrm{^{\prime}}}& {H}_{22}^{*\mathrm{^{\prime}}}& {H}_{23}^{*\mathrm{^{\prime}}}& {H}_{24}^{*\mathrm{^{\prime}}}& {H}_{25}^{*\mathrm{^{\prime}}}& {H}_{26}^{*\mathrm{^{\prime}}}& {H}_{27}^{*\mathrm{^{\prime}}}\\ {H}_{31}^{*\mathrm{^{\prime}}}& {H}_{32}^{*\mathrm{^{\prime}}}& {H}_{33}^{*\mathrm{^{\prime}}}& {H}_{34}^{*\mathrm{^{\prime}}}& {H}_{35}^{*\mathrm{^{\prime}}}& {H}_{36}^{*\mathrm{^{\prime}}}& {H}_{37}^{*\mathrm{^{\prime}}}\\ {H}_{41}^{*\mathrm{^{\prime}}}& {H}_{42}^{*\mathrm{^{\prime}}}& {H}_{43}^{*\mathrm{^{\prime}}}& {H}_{44}^{*\mathrm{^{\prime}}}& {H}_{45}^{*\mathrm{^{\prime}}}& {H}_{46}^{*\mathrm{^{\prime}}}& {H}_{47}^{*\mathrm{^{\prime}}}\\ {H}_{51}^{*\mathrm{^{\prime}}}& {H}_{52}^{*\mathrm{^{\prime}}}& {H}_{53}^{*\mathrm{^{\prime}}}& {H}_{54}^{*\mathrm{^{\prime}}}& {H}_{55}^{*\mathrm{^{\prime}}}& {H}_{56}^{*\mathrm{^{\prime}}}& {H}_{57}^{*\mathrm{^{\prime}}}\\ {H}_{61}^{*\mathrm{^{\prime}}}& {H}_{62}^{*\mathrm{^{\prime}}}& {H}_{63}^{*\mathrm{^{\prime}}}& {H}_{64}^{*\mathrm{^{\prime}}}& {H}_{65}^{*\mathrm{^{\prime}}}& {H}_{66}^{*\mathrm{^{\prime}}}& {H}_{67}^{*\mathrm{^{\prime}}}\\ {H}_{71}^{*\mathrm{^{\prime}}}& {H}_{72}^{*\mathrm{^{\prime}}}& {H}_{73}^{*\mathrm{^{\prime}}}& {H}_{74}^{*\mathrm{^{\prime}}}& {H}_{75}^{*\mathrm{^{\prime}}}& {H}_{76}^{*\mathrm{^{\prime}}}& {H}_{77}^{*\mathrm{^{\prime}}}\end{array}\right].$$

The amplitude displacement vector corresponding to each path is given by Eq. (21).

21 $${{\varvec{Q}}}^{*\mathrm{^{\prime}}}=\left\{\begin{array}{ccccccc}{\Xi }_{s1,g1}^{*}& {\Xi }_{s2,g1}^{*}& {\Xi }_{s3,g1}^{*}& {\mathrm{\wp }}_{st}^{*}& {\Xi }_{s1,g2}^{*}& {\Xi }_{s2,g2}^{*}& {\Xi }_{s3,g2}^{*}\end{array}\right\}$$

To calculate the amplitude corresponding to each path, Eq. (22) is used.

22 $${{\varvec{Q}}}^{*\mathrm{^{\prime}}}{e}^{i\omega t}={{\varvec{H}}}^{*\mathrm{^{\prime}}}\left\{{{\varvec{F}}}^{*}+{{\varvec{W}}}^{*}\right\}{e}^{i\omega t}$$

The amplitude is defined as in Eq. (23), and the phase can be defined as in Eq. (24) based on the amplitude:

23 $${\Xi }_{si,1}^{*}=({H}_{i1}^{*\mathrm{^{\prime}}}+{H}_{i4}^{*\mathrm{^{\prime}}}d+{H}_{i6}^{*\mathrm{^{\prime}}}d){W}_{z},{\Xi }_{si,st}^{*}={H}_{i3}^{*\mathrm{^{\prime}}}{F}_{st}$$

24 $${\beta }_{si,1}=\mathrm{\angle }({H}_{i1}^{\mathrm{^{\prime}}}+{H}_{i4}^{\mathrm{^{\prime}}}d+{H}_{i6}^{\mathrm{^{\prime}}}d),{\beta }_{si,st}=\mathrm{\angle }{H}_{i3}^{\mathrm{^{\prime}}}$$

where $\mathrm{\angle }$ represents the phase operator, and ${\beta }_{si,1}$ and ${\beta }_{si,st}$ represent the phase caused by the disturbance and actuator force, respectively. If Eq. (19) is redefined for magnitude and phase, it can be rewritten as

25 $${\xi }_{i}^{*}\left(t\right)=\left(\left|{\Xi }_{si,1}^{*}\right|{e}^{i{\beta }_{si,1}}+\left|{\Xi }_{si,st}^{*}\right|{e}^{i\left({\beta }_{si,st}+{\phi }_{st}\right)}\right){e}^{i\omega t}.$$

Equation (25) has three phases, i.e., two phases generated by the disturbance and actuator force, and one phase generated by the actuator; that makes controlling the motion of the actuator difficult. To perform motion control conveniently, phase matching is conducted for the phase generated by the disturbance force. The phase generated by the disturbance force can be assumed to be equal to the sum of the phases generated by the actuator. For example, expressing the part of path1 can be written as ${\beta }_{s\mathrm{1,1}}={\beta }_{s1,st}+{\phi }_{st}$. Through this assumption, the actuator phase can be defined as

26 $${\phi }_{st}={\beta }_{si,1}-{\beta }_{si,st}.$$

Using Eq. (26), the out-of-phase occurring in the *i*th path can be generated, and the *i*th source motion can be summarized as

27 $${\xi }_{i}^{*}\left(t\right)=\left(\left|{\Xi }_{si,1}^{*}\right|+\left|{\Xi }_{si,st}^{*}\right|\right){e}^{i\left(\omega t+{\beta }_{si,1}\right)}.$$

To isolate the vibration for the target path, the actuator force ${F}_{st}$ is calculated by assuming zero amplitude:

28 $$\left|{\Xi }_{si,1}^{*}\right|+\left|{\Xi }_{si,st}^{*}\right|=0.$$

Using Eq. (28), the ${F}_{st}$ can be expressed as follows:

29 $$\left|{H}_{33}^{*}\right|\left\{{F}_{st}\right\}=-{W}_{z}\left|{H}_{31}^{*}+d{H}_{34}^{*}+d{H}_{36}^{*}\right|.$$

## Numerical performance validation with a single sinusoid

### Simulation overview

A simulation was performed to validate the mathematical model. It was expressed through the state-space equation, which is summarized as follows:

30 $${\dot{x}}^{\mathrm{^{\prime}}}(t)={\varvec{A}}{{\varvec{x}}}^{\mathrm{^{\prime}}}(t)+{\varvec{B}}{\varvec{u}}(t)$$

31 $${y}^{\mathrm{^{\prime}}}(t)={\varvec{C}}{{\varvec{x}}}^{\mathrm{^{\prime}}}(t)+Du(t)$$

In Eqs. (30) and (31), A, B, and C represent the matrices corresponding to the state of the system, input, and output, respectively, and are given in Eqs. (32)–(35). In addition, the output $y(t)$ is defined as having six outputs for three source parts and three receiver parts, and is given by Eq. (36). In Eq. (36), ${a}_{pn,gm}$ represents the acceleration corresponding to the *n*th path in the mass with the *m*th inertia.

32 $${\varvec{A}}=\left[\begin{array}{cc}{O}_{n\times n}& {I}_{n\times n}\\ -\frac{{{\varvec{K}}}^{\mathrm{^{\prime}}}}{{{\varvec{M}}}^{\mathrm{^{\prime}}}}& -\frac{{{\varvec{C}}}^{\mathrm{^{\prime}}}}{{{\varvec{M}}}^{\mathrm{^{\prime}}}}\end{array}\right],$$

33 $${\varvec{B}}=\left[\begin{array}{c}{O}_{n\times n}\\ \frac{1}{{{\varvec{M}}}^{\mathrm{^{\prime}}}}\end{array}\right]$$

34 $${\varvec{C}}=\left[\begin{array}{ccc}{I}_{3\times 3}& {O}_{3\times 3}& {O}_{3\times 3}\\ {O}_{3\times 3}& {O}_{3\times 3}& {I}_{3\times 3}\end{array}{O}_{9\times 9}\right],$$

35 $${\varvec{D}}=\left[{O}_{6\times 9}\right]$$

36 $$y(t)={\left\{{a}_{p1,g1}{a}_{p2,g1}{a}_{p3,g1}{a}_{p1,g2}{a}_{p2,g2}{a}_{p3,g2}\right\}}^{T}$$

### Control results by the quantification method

The simulation was performed based on the state-space equation discussed in “Simulation overview” section. In addition, the quantification of the actuator force and phase determined in “Quantification of actuator input” section was used as the actuator input signal. The disturbance and actuator forces are defined in Eqs. (37) and (38), where $\omega$ is 460 Hz.

37 $$u(t)=10\mathit{sin}(\omega t)$$

38 $${f}_{st}(t)={F}_{st}\mathit{sin}(\omega t+{\phi }_{st})$$

**Figure 3:**
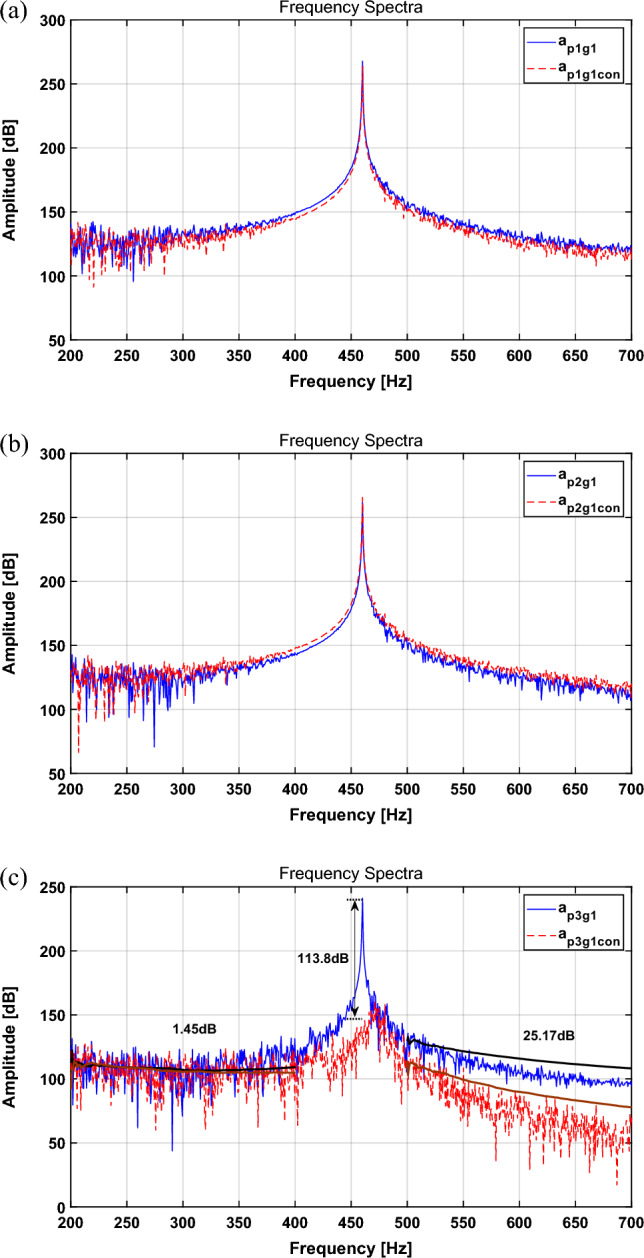
Spectrum comparison for a sinusoid with quantification method: (**a**) path 1, (**b**) path 2, (**c**) path 3. Key: (blue solid line) actuator turned off; (red dashed line) actuator turned on.

The sampling frequency was set to 15 kHz, and the simulation results are expressed in Fig. 3. The RMS and peak values are summarized in Table 2.

**Table 2:** Change in the broadband RMS value for each path and main frequency peak with the quantification method.

Case	Broadband RMS [dB]			Main freq. peak [dB]		
	Path 1	Path 2	Path 3	Path 1	Path 2	Path 3
Actuator turned off	141.87	136.02	117.09	267.7	261.7	241.1
Actuator turned on	138.18	140.12	99.24	263.9	265.8	127.3
Difference	− 3.7 (2.6%↓)	+ 4.1(3.0%↑)	− 17.9(15.3%↓)	3.8 dB↓	4.1 dB↑	113.8 dB↓

In Table 2, the spectral results show a tendency to decrease by 2.6% and 15.25% for paths 1 and 3, respectively, and increase by 3.01% for path 2. Table 2 presents a comparison of the results of the main frequency attenuation. For path 1, the main frequency attenuation tends to decrease by approximately 3.8 dB before and after control, and that for path 2 tends to increase by 4.1 dB, while that for path 3 tends to decrease by approximately 113.8 dB.

Because the actuator force calculated in “Quantification of actuator input” section targets the perfect isolation of the active path (path 3), it is desirable to observe the best attenuation in path 3. In addition, for paths 1 and 2, the changes in the RMS and peak value tend to be rather small compared to those of path 3, even though path 2 shows a slight amplification. This is because it is located near the shaker, and the effect of the active path seems to degrade. Based on the above results, the vibration isolation performance of the targeted path is excellent; however, there are some limitations: (1) When the quantified force and phase of the actuator are applied, they show good vibration isolation performance for the targeted part, but they cannot guarantee vibration reduction in other paths; (2) it is inconvenient to calculate the force and phase each time the structure is changed; (3) there is little attenuation in broadband; and (4) the force and phase can be obtained only for a sinusoidal signal, which means that they are difficult to apply to complex signals that occur in real life. Therefore, in order to overcome these limitations, active vibration control was performed by applying the NLMS algorithm.

### Multi-NLMS algorithm

**Figure 4:**
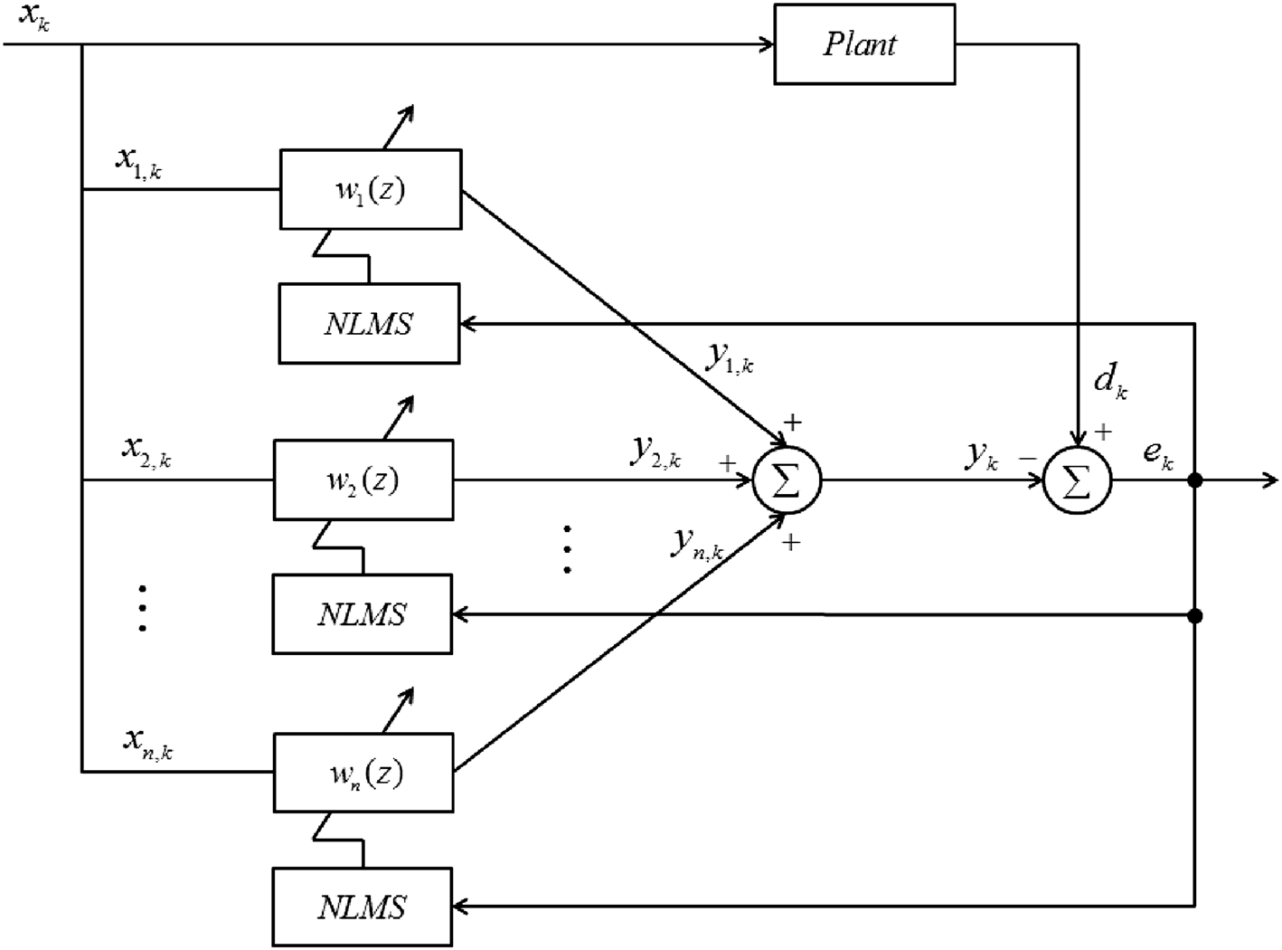
Schematic for the Multi-NLMS algorithm.

The vibration signal generated by an electric motor has a complex spectrum with at least three or more fundamental frequencies. Because it is difficult to adopt the quantification method described in “Quantification of actuator input” section, it is preferable to apply an adaptive digital filter for the reduction control of vibration signals. These classify the iterative algorithm, which updates the filter weights based on the errors between the plant and filter signals. Through this process, the filter can follow the plant signal. Thus, the adaptive digital filter is utilized for structural analysis and control because it has the ability to generate the desired signal among those occurring in the system. Among numerous digital filters, in this study, the NLMS algorithm was adopted and used for vibration reduction. Since NLMS has a simple structure, it has the advantage of being easy to implement. In particular, it has excellent performance in tracking signals compared to simple structures, so it is widely applied in many industrial fields to reduce plant vibration and noise. Furthermore, in order to track signals with multiple frequencies, multiple NLMS algorithms are actively used. Thus, in this study, control was performed by applying a basic NLMS for sinusoidal signals and a multi-NLMS with three channels for AM signals. A schematic of the multi-NLMS is shown in Fig. 4.

Here, ${x}_{k}$ and ${d}_{k}$ represent the signals corresponding to the disturbance and the plant output, respectively. ${y}_{k}$ represents the signal combining each filter output and ${e}_{k}$ represents the error signal between ${d}_{k}$ and ${y}_{k}$. The weight update equation for the NLMS algorithm is

39 $${\varvec{W}}\left(k+1\right)={\varvec{W}}\left(k\right)+\frac{\mu }{{\Vert X\left(k\right)\Vert }^{2}+\delta }{\varvec{X}}\left(k\right)e\left(k\right).$$

In Eq. (39), $\mu$ and $\delta$ represent the step size and safety factor (small positive constant), respectively. The output from the multi NLMS tracks the plant signal and was used as the input signal of the actuator to perform vibration control.

### Control results with NLMS algorithm

**Figure 5:**
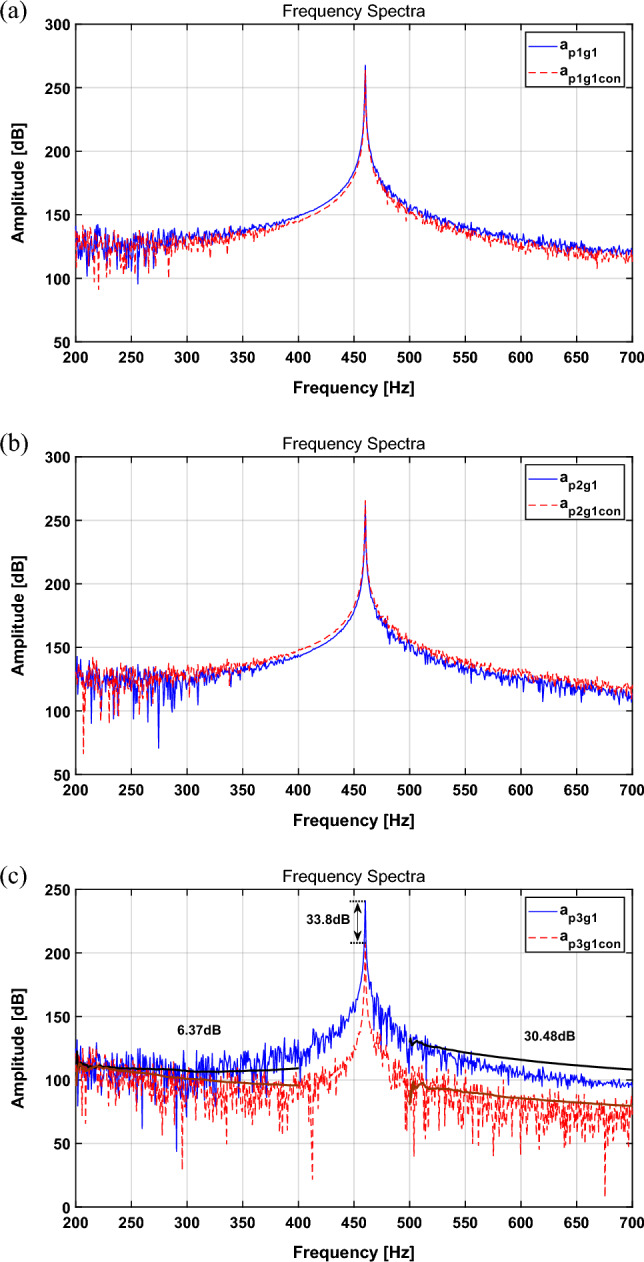
Spectrum comparison for a sinusoid with NLMS algorithm: (**a**) path 1, (**b**) path 2, (**c**) path 3. Key: (blue solid line) actuator turned off; (red dashed line) actuator turned on.

To compare the performance of the quantification method and the NLMS algorithm, simulations were performed under the same conditions. The disturbance force $u(t)$ was used, and the sampling frequency was set to 15 kHz. The actuator input signal was applied using the NLMS output through appropriate adjustment. The simulation results are shown in Fig. 5 and are summarized in Table 3.

**Table 3:** Change in the broadband RMS value for each path and main frequency peak with the NLMS algorithm.

Case	Broadband RMS [dB]			Main freq. peak [dB]		
	Path 1	Path 2	Path 3	Path 1	Path 2	Path 3
Actuator turned off	141.87	136.02	117.09	267.7	261.7	241.1
Actuator turned on	139.05	139.30	93.82	264.6	265.2	207.3
Difference	− 3.34 (1.99%↓)	+ 1.44 (2.41% ↑)	− 13.97 (19.87%↓)	3.1 dB ↓	3.5 dB ↑	33.8 dB ↓

In Table 3, comparing the uncontrolled and controlled RMS values for the spectrum, those of paths 1 and 3 show a tendency to decrease by 1.99% and 19.87%, respectively, while those of path 2 tend to increase by 2.41%. Table 3 presents a comparison of the results of the main frequency attenuation. For paths 1 and 3, the main frequency attenuation tends to decrease by 3.1 dB and 33.8 dB, respectively, while that of path 2 tends to increase by 3.5 dB after control is applied. In addition, the broadband attenuation when the quantification method and NLMS algorithm are also observed. SBB represents the sinusoidal broadband. First, in the case of SBB1, the quantification method reduces it by 1.45 dB, while it is reduced by 6.37 dB when the NLMS is applied. Moreover, the SBB2 tends to be decreased by 25.17 dB and 30.48 dB, respectively.

A comparison of the results between the quantification method and the NLMS algorithm shows the following differences. The isolation performance for the primary frequency is better with the quantification method, whereas the effect of broadband attenuation is better with the NLMS algorithm. From the point of view of attenuating the overall system vibration, it is important to reduce the primary frequency, but broadband attenuation is also an important part of the overall vibration attenuation. Therefore, the above results prove that applying the NLMS algorithm is more effective in attenuating the overall system vibration. Furthermore, the signal generated by the actual electric motor generates a multi-frequency signal containing several frequencies. However, when a single NLMS algorithm is applied, it has limitations in tracking multi-frequency signals. Therefore, in the next chapter, multi-NLMS algorithm was applied and simulated for multi-frequency signal control.

## Numerical performance validation with a multi-spectral signal

To validate the reducing performance of the vibration for the multi-frequency, the AM signal given by Eq. (40) is used. For the AM signal, the path 3 signal from the source is tracked through a multi-NLMS with three channels. During the simulation, the sampling frequency was set to 15 kHz.

40 $$u\left(t\right)=\left(1+0.5\mathrm{cos}\left(2\pi \cdot 140t\right)\right)\times 5\mathrm{sin}(2\pi \cdot 460t)$$

**Figure 6:**
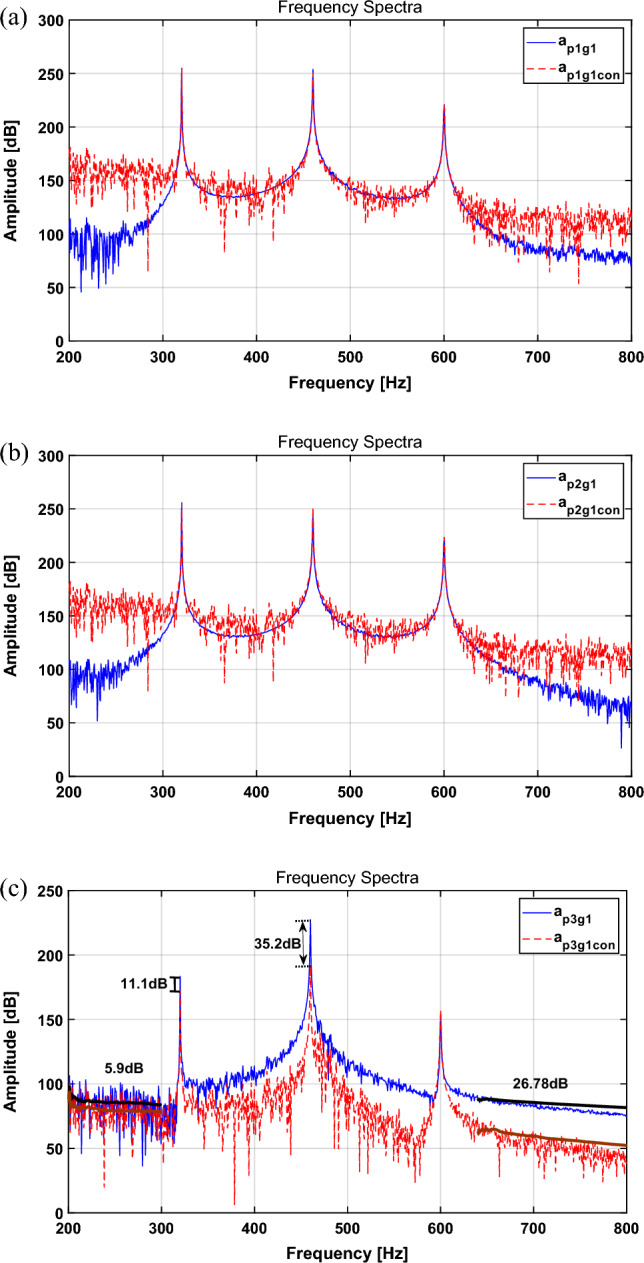
Spectrum comparison for AM signal with Multi-NLMS algorithm: (**a**) path 1, (**b**) path 2, (**c**) path 3. Key: (blue solid line) actuator turned off; (red dashed line) actuator turned on.

The actuator input signal was applied using the multi-NLMS output through appropriate adjustment. The simulation results for each path and the decibel reduction rates for path 3 are summarized in Table 4. The results for the frequency domain are shown in Fig. 6.

**Table 4:** Change in the broadband RMS value for each path and the main frequency of AM signal in path 3 with the multi-NLMS algorithm.

Case	Broadband RMS [dB]			Main freq. peak [dB]		
	Path 1	Path 2	Path 3	320 Hz	460 Hz	600 Hz
Actuator turned off	133.27	132.78	102.77	183.5	227.3	153.7
Actuator turned on	146.02	147.18	82.97	172.4	192.1	156.9
Difference	+ 12.75 (9.57%↑)	+ 14.40 (10.84%↑)	− 19.80 (19.27%↓)	11.1 dB ↓	35.2 dB ↓	3.2 dB ↑

In Table 4, when comparing the uncontrolled and controlled RMS values for the spectrum, those of paths 1 and 2 show a tendency to increase by 9.57% and 10.84%, respectively. However, in the case of path 3, they show a tendency to decrease by 19.27%. The difference between uncontrolled and controlled results shows a decrease of 11.1 dB and 35.2 dB for 320 Hz and 460 Hz, respectively. In addition, at 600 Hz, it shows a tendency to increase by 3.5 dB. ABB represents the AM broadband signal. In the case of ABB1, it shows a decrease of 5.9 dB, and ABB2 shows a decrease of 26.78 dB, which tends to be similar to that obtained for the sinusoid in “Control results with NLMS algorithm” section.

When the AM signal was applied as a disturbance force, the vibration reduction performance was verified by applying a multi-NLMS algorithm. Because only path 3 has a piezoelectric stack actuator, its vibration reduction performance is superior to that of the other two paths. The RMS results of paths 1 and 2 tend to increase, which appears to be the result of additional external forces from the active path. On the other hand, in the case of path 3, it can be seen that the vibration is greatly reduced at a primary frequency. Furthermore, the attenuation performance of broadband showed a tendency to decrease by 5.9 and 26.78 dB. A comprehensive result proved that it exhibited excellent vibration attenuation performance when active vibration control was performed by applying a piezoelectric stack actuator to the path. Moreover, based on the above results, if an actuator is installed in all paths, it is expected that vibrations for the entire path can be reduced by carefully considering the interactions among paths, which will be a scope for future research.

## Experimental validation

### Setup configuration

**Figure 7:**
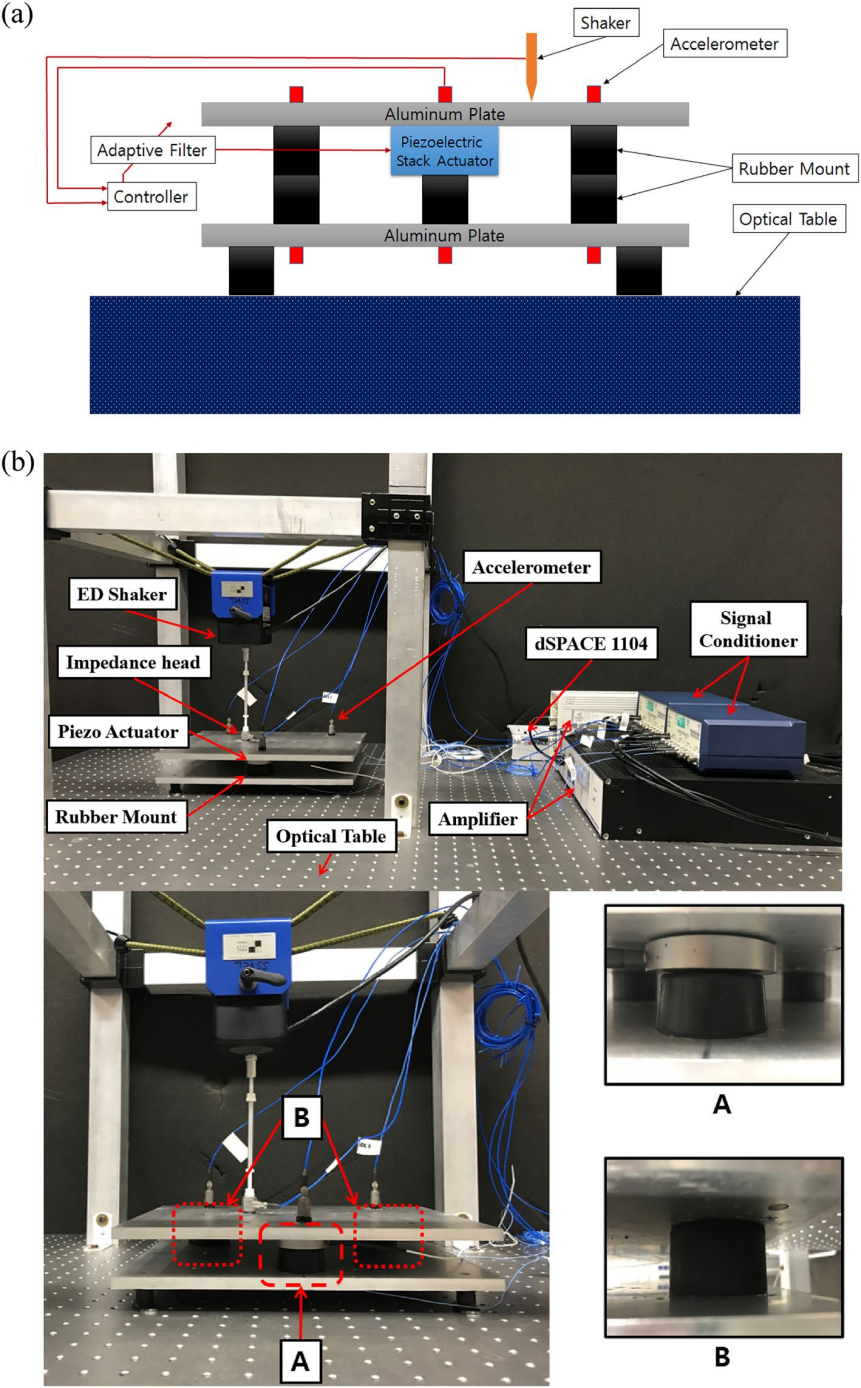
(**a**) Schematic of the experimental setup, (**b**) experimental set-up for the 7 DOF structure/composition of each path.

To validate the simulation results presented in the previous sections, a feasibility experiment was conducted using a laboratory setup with a plate-like structure. A schematic of the experimental setup is shown in Fig. 7a. The source and receiver, which had a plate shape, were made of aluminum, as shown in Fig. 7b. These had a thickness of 10 mm and length of 240 × 330 mm. In addition, a path existed between the source and receiver, which consisted of two passive paths and one active path. The passive path consisted only of a rubber grommet, and the active path consisted of a piezoelectric stack actuator and rubber grommet. To perform the source motion control, the accelerometer signal was tracked for the active path through the NMLS and multi-NLMS algorithms, and it was used as the input signal for the piezoelectric stack actuator. The composition of each path is shown in Fig. 7b, which shows that paths 1 and 2 consist only of a rubber grommet, and path 3 consists of a rubber grommet and a piezoelectric stack actuator. When the experiment was performed, the accelerometer signal was measured in real time using dSPACE 1104, which is a real-time control protocol. In addition, a disturbance force was applied using an electrodynamic shaker, and it was measured using the impedance head attached to the end of the stinger. This study focused on source motion control through the active path; thus, the receiver signals were ignored.

Prior to the experiment, the alignment of the stinger between the source and shaker was matched, and the resonance region for the structure was checked. To check the alignment and resonance, the response of the experimental structure was obtained through an impact test and the chirp signal. The natural frequencies of the experimental setup are 40, 390, 480, and 950 Hz.

### Experimental results with a sinusoid

**Figure 8:**
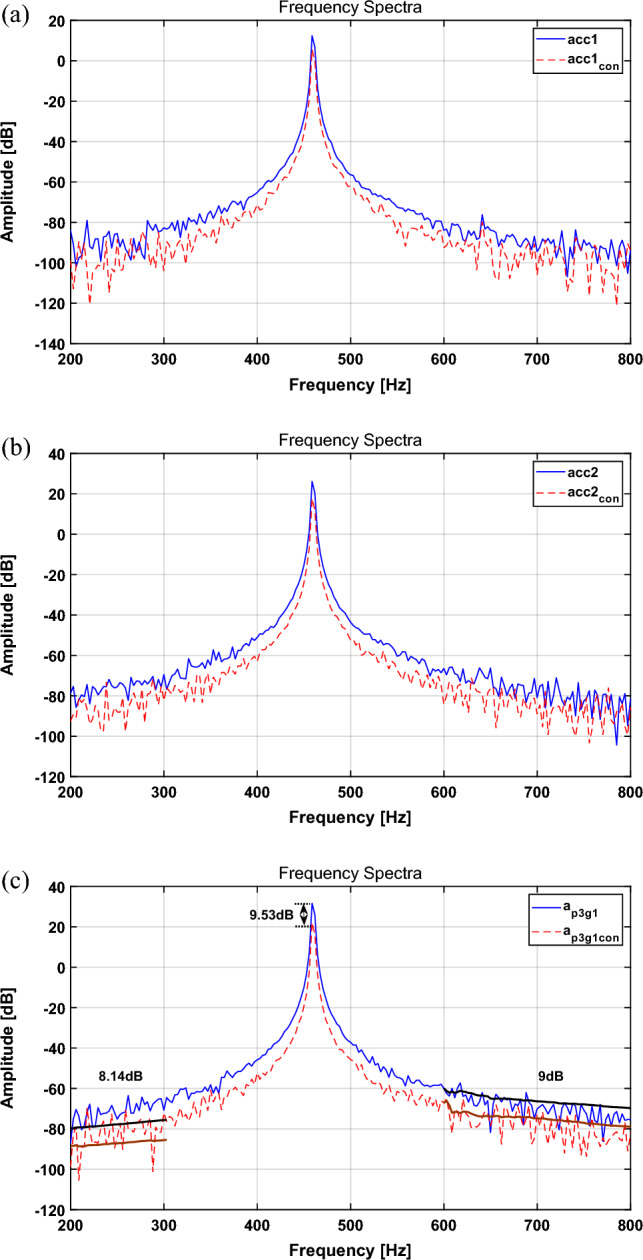
Spectrum comparison for a sinusoid with the NLMS algorithm: (**a**) path 1, (**b**) path 2, (**c**) path 3. Key: (blue solid line) actuator turned off; (red dashed line) actuator turned on.

To validate the results of the numerical simulation through a feasibility experiment, the disturbance force was applied by a sinusoidal signal defined in “Control results by the quantification method” section. In addition, the source signal of path 3 was tracked through the NLMS algorithm, and it was used as an actuator input signal through proper adjustment. The experimental results are shown in Fig. 8. The RMS values and the reduction performance of the primary frequency is summarized in Table 5.

**Table 5:** Change in the broadband RMS value for each path and the change in the main frequency with the NLMS algorithm (sinusoid, experiment).

Case	Broadband RMS [dB]			Main freq. peak [dB]		
	Path 1	Path 2	Path 3	Path 1	Path 2	Path 3
Actuator turned off	− 81.76	− 69.31	− 63.55	12.39	26.09	31.64
Actuator turned on	− 89.52	− 77.40	− 72.51	5.52	17.56	22.11
Difference	− 7.76 (8.67% ↓)	− 10.45 (10.45%↓)	− 8.96 (12.36% ↓)	6.87 dB ↓	8.53 dB ↓	9.53 dB ↓

The spectral results in Table 5 show that the broadband RMS value paths 1, 2, and 3 tend to decrease by 8.67%, 10.45%, and 12.36%, respectively. In Table 5, the main frequencies for paths 1, 2, and 3 show a tendency to decrease by about 6.87, 8.53, and 9.53 dB, respectively, for uncontrolled and controlled results. This result indicates that the reduction rate of path 3 was superior to those of the other paths. In addition, the broadband attenuation rate reveals that the attenuation tendency decreased by approximately 9 dB. Compared with the simulation results in “Setup configuration” section, the results reveal a proper correlation between the primary frequency and broadband vibration attenuation. In addition, the attenuation effect can be affected by applying the active mounting system to one path.

### Experimental results with an amplitude modulated signal

**Figure 9:**
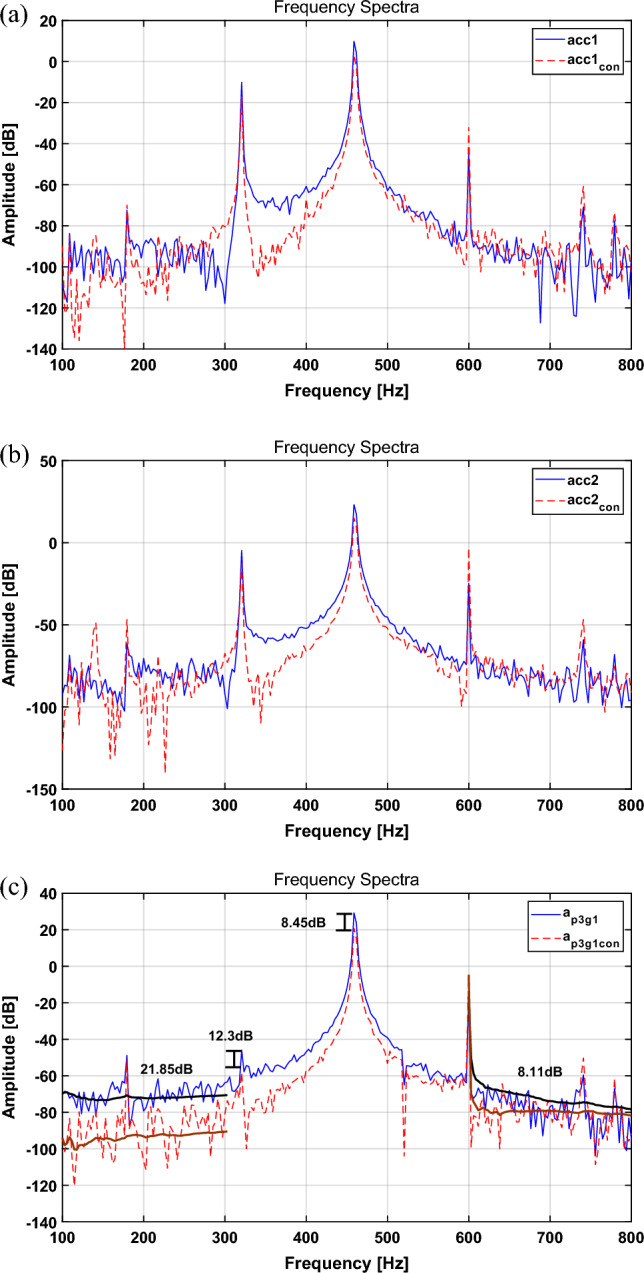
Spectrum comparison for the AM signal with the multi-NLMS algorithm: (**a**) path 1, (**b**) path 2, (**c**) path 3. Key: (blue solid line) actuator turned off; (red dashed line) actuator turned on.

To validate the results of the numerical simulation through a feasibility experiment, the disturbance force was applied using the AM signal defined in “Numerical performance validation with a multi-spectral signal” section. In addition, the source signal of path 3 was tracked through a multi-NLMS algorithm with three channels, and it was used as an actuator input signal through proper adjustment. The experimental results are shown in Fig. 9. The RMS values and the reduction performance of the primary frequency is summarized in Table 6.

**Table 6:** Change in the broadband RMS value for each path and the change in the main frequency with the multi-NLMS algorithm (AM signal, experiment).

Case	Broadband RMS [dB]			Main freq. peak [dB]		
	Path 1	Path 2	Path 3	320 Hz	460 Hz	600 Hz
Actuator turned off	− 85.09	− 72.35	− 65.43	− 45.95	29.25	− 16.76
Actuator turned on	− 87.86	− 77.14	− 76.07	− 58.25	20.8	− 4.71
Difference	− 2.77 (3.15%↓)	− 4.79 (6.21%↓)	− 10.64 (13.99%↓)	12.3 dB ↓	8.45 dB ↓	12.1 dB ↑

Comparing the uncontrolled and controlled results for the time domain in Table 6, the results for the spectrum tend to decrease by approximately 3.15%, 6.21%, and 13.99%, respectively, for paths 1, 2, and 3. When comparing the frequency reduction performances in Table 6, the reduction tends to decrease by 12.3 and 8.45 dB at 320 and 460 Hz, respectively. However, at 600 Hz, it shows a tendency to increase by 12.1 dB. Furthermore, the broadband attenuation performance decreases by 21.85 dB in the 100–300 Hz band and 8.11 dB in the 600–800 Hz band. Compared with that achieved with the simulation in “Numerical performance validation with a multi-spectral signal” section, the attenuation effect in broadband is slightly different; however, the trend for the overall reduction performance is similar. In addition, the results reveal that reduction in the entire path not shown in the simulation is possible. Based on these results, it seems that efficient source motion control is possible when actuators are present in the entire path.

## Conclusion

This study focused on source motion control by applying an active mounting system with a piezoelectric stack actuator. The vibration attenuation performance was verified through numerical simulations and feasibility experiments. Modeling of the entire structure with an active path was performed based on a lumped parameter model. The three paths consisted of two passive paths with a rubber grommet and one active path with a piezoelectric stack actuator and rubber grommet in series. Furthermore, to perform active control, an adaptive filter was applied with NLMS and multi-NLMS algorithms. Source motion control was achieved when sinusoidal and AM signals were used as the disturbance forces. Through numerical simulations and feasibility experiments, the vibration in the active path with an active mounting system was effectively reduced.

The main contributions of this study are as follows: (1) The force and phase of the active mounting system were calculated using a quantification method; (2) a simulation was performed using quantified values and the NLMS or multi-NLMS algorithm, and these results were compared to validate the vibration attenuation performance; and (3) a feasibility experiment was performed to demonstrate the vibration attenuation performance of an active mounting system. In the quantification method, the force and phase of the actuator were calculated when the sinusoid was used as the disturbance force. Using these values, the vibration-isolation performance for the targeted path was validated. However, this method has several limitations: (1) The force and phase can be obtained only for a sinusoidal signal, which means that they are difficult to apply to complex signals that occur in real life; (2) it is inconvenient to change the structure; and (3) as the degree of freedom increases, the amount of computation increases, which limits the application of this method in real life. To overcome these limitations, source motion control was performed using an NLMS or a multi-NLMS algorithm. The vibration reduction of the quantification method appeared to be higher than that of the NLMS algorithm at the primary frequency because the quantification method was performed with the assumption of an isolated magnitude, but the method of applying NLMS had a higher broadband reduction rate. Furthermore, to consider a more complex signal, the AM signal was used as the disturbance force, and the multi-NLMS algorithm with three channels was applied to the control. The results reveal that the primary and sideband frequencies are significantly reduced through the active mounting system. To verify the performance of vibration attenuation, a feasibility experiment was conducted using the principle experimental setup. This proves that the results of the numerical simulation and feasibility experiment exhibit an appropriate correlation.

In future research, simulations and experiments will be performed with the active mount system installed in all paths. In addition, the interaction between each path will be investigated to achieve optimal control. Practical modeling should be performed by considering the position of the mount attached to the actual engine.

## Data Availability

The datasets used and/or analysed during the current study available from the corresponding author on reasonable request.
